# A Study of the Diseases of the Peridental Membrane, Having Their Origin at or near the Gingival Margin

**Published:** 1893-05

**Authors:** H. A. Kelley

**Affiliations:** Portland, ME.


ARTICLE IV.

### A STUDY OF THE DISEASES OF THE PERIDENTAL MEMBRANE, HAVING THEIR ORIGIN AT OR NEAR THE GINGIVAL MARGIN.

BY H. A. KELLEY, D. M. D., PORTLAND, ME.

Read before the Maine Dental Society.

The condition I am about to consider to-night is most familiarly known to you, no doubt, as that of Riggs' disease; but it behooves us to use professional language and methods. It is the aim of science to obtain as perfect a nomenclature as possible, and it is not proper to name a disease after its demonstrator, however much we may wish to honor him. But I do not think any one name can designate the conditions. There are many under this one head, seemingly the same and yet very different; having variable symptoms and exciting causes. It seems to me much of the difference of belief in earlier times and even to-day in regard to this disease was and is due to this fact. These have not yet been classified, but are regarded as one pathological condition. Nothing but confusion can result from this, and it is not strange to find many theories in regard to it, and especially as to its origin.

It is the purpose of this paper to give certain conclusions of mine, in hopes that I may contribute something that may aid us to a clearer standing of a disease so destructive in its results and yet so little understood.

Salivary calculus, I think all will agree, is not the cause of serious disease. It is deposited at or near the salivary ducts at the gingival margin or just above it, towards the cutting edge of the tooth. The theory of this precipitation is as follows: The saliva contains both calcium phosphate and calcium carbonate, and these are held in solution by carbonic acid before the saliva is poured into the mouth. As the saliva enters the mouth this carbonic acid escapes and the lime salts are precipitated. This may be shown experimentally by a bottle with these salts in carbonic acid solution; allow acid to escape and salts will be precipitated. As the calculus pushes the gum down new deposits form and as a result the gum may lose its tone and thus a more or less serious state of affairs result. But I think there is no attempt of this kind of calculus to work in under the gum margin, and only by the greatest neglect can it do serious harm.

It is easily scaled away, and when so removed we find the gum underneath it in practically a healthy state, only a more or less loss of tone being seen as there has been more or less neglect. The gum soon returns to its normal condition after its removal.

The results, possibly caused by serumic or sanguinary calculus, must be considered. First, we do not know this species of calculus comes from the serum, or even from the blood. It is only a belief based upon certain analyses. "There is no evidence that it is derived from the blood. It may possibly be deposited from the pus bathing the tooth and more probably from the saliva." (Truman.)

It certainly is different from ordinary tartar, and much more destructive in its results. Salivary calculus is a semi-solid mass, composed chiefly of water, calcium phosphate, and calcium carbonate, with a trace of calcic fluoride and magnesium phosphate. It is easily scaled away, as it does not seem to firmly adhere to the tooth as does the serumic. The latter, on the contrary, is a very hard substance, and removed only with great difficulty. It has not been analyzed, and I cannot correctly give you its constituents. What relation does this calculus bear to the disease?

In one of the latest papers on this subject, Dr. Allan says: "To me it always seems that much of the trouble that many dentists meet . . . arises from a total misconception of its origin and cause . . . I desire to state positively my helief that pyorrhœa alveolaris is always preceded by a deposit of serumal tartar." This statement he enforces later by saying, "If I had said, as a rule, the so-called pyorrhœa alveolaris had its origin in a purely local cause or causes, and that nine out of ten times this local cause was tartar in some of its protean forms, I would have rightly stated my opinion. I go further and say the constitutional diathesis theory begs the question and cannot stand close examination. All that can be said for a systemic origin is, the inflamed gingival or mucous membrane is prone to secrete lime salts, and these salts, in turn, become added causes of irritation." Dr. Allan believes the healthy mucous glands do not secrete tartar, and he regards a natural or acquired roughness of the neck of the tooth as the exciting cause of the unhealthy action of the glands that induces them to secrete the tartar. Dr. Allan says, while strongly of the opinion that this is correct, he is not sure of his position.

These are strong words and fill us full of hope that Dr. Allan has at last settled something. But this hope is quite dispelled, and we find ourselves again in doubt when, in the discussion of this paper, we hear the following from Dr. Truman: "Now, what is the origin of this pathological condition? Does it originate from tartar? Not if I understand it. Does it originate from the roughness of the gingival border of the teeth that Dr. Allan mentioned? When you find a bright red line at the border-line of the gum, there is the beginning. It has nothing to do with tartar. It may come from constitutional disturbances; it may originate from some form of nephritis, or a long siege of sickness. Immediately succeeding we have a development of micro-organic life. This disease has its origin in inflammation of the periosteum or pericementum of the roots of the teeth. There is no question about that. It naturally follows if we are to treat the teeth properly we must direct our attention to the micro-organic life first, and not to the tartar, which is secondary. Where tartar is, in my judgment, there cannot arise—does not arise—this pathological condition." Dr. Sudduth agrees that tartar is secondary to the disease, but says, "The initial phase we do not know. No man has ever been able to tell the cause of the disease; there is a catarrhal process, but what causes that to be set up has not as yet been solved. The deposits on the roots is a result of this catarrhal process. First, there is irritation, then follows micro-organisms, and these, in turn, become a source of irritation; but their direct connection with the disease has not been determined."

Many of us think the very worst cases we have ever seen were entirely devoid of tartar, either serumal or any other kind. Now, it seems to me, in this disease, accompanied by deposits of tartar, we have what I consider one of two conditions that are described by various authors by the same name; and it also seems that whatever may be the predisposing cause, the exciting cause is the so-called serumal tartar.

In support of this statement I fear I can but repeat the old reasons for and against the local causation of the disease, but hope to be able to present them in a way that will convince you of their truth. It is my belief, as stated by Dr. Allan, that those persons that support the systemic theory only beg the question. It is so satisfying to throw the disease back on to the system. But what reason have we for so doing? We have a pathological state here, always accompanied by calculus, which may be cured by the simple removal of the calculus, nothing else being necessary. The only way it can be treated is by the complete removal of this, and by so much as you thoroughly accomplish that you will succeed. Could anything point more clearly to the cause of the disease? Systemic conditions are but side issues; important, but not the origin. In support to this statement I need to present but the one fact, a complete removal of the calculus will effect a cure. Not one case is on record of failure where this was thoroughly done. If the system was at fault there would be exceptions to this rule. Dr. W. D. Miller says, "My view is, three factors must be taken into consideration in each case of pyorrhœa alveolaris,—first, predisposition; second, local irritation; third, fungi."

I pass now to the last form of the conditions we are studying, the condition called phagedenic pericementitis. Many have adopted this name to designate all the lesions we have been considering. I prefer to use it as descriptive of this one condition. This, then, as the term implies, is an inflammation of the pericementum, resulting in rapid ulceration. The gums are but slightly affected. Thin flat instruments can be passed farther up the root than is possible in a normal condition. The destruction of the tissues of the peridental membrane has begun, and the pockets, that I mentioned as indicative of the disease, are forming.

This destructive process proceeds in the direction of the fibres of the membrane,—that is, towards the apex of the root. It may affect but one side of the root, and there may be pockets with healthy membrane between; but usually the affected tract widens and destroys the entire root membrane. Although it seems to be an infectious disease, the fact is not fully established. There is little or no recession of the gum and the condition may be overlooked except a very careful examination be made. As a result of the loss of the membrane, the alveolar process disappears.

I have thus briefly described the simplest form of phagedenic pericementitis, but by far the greater number of cases are complications of this disease and due to calcic inflammation.

I have said of this form I believed the exciting cause to be calculus, but as the disease may certainly begin and run its course without any deposition of calculus, we must turn elsewhere for the origin. And it seems highly probable it is due to fungi or some form of micro-organism, and as I have said is, or seems to be, infectious. Dr. Black thought, at one time, he had isolated the fungus; but further investigation did not confirm his hopes. Miller has not been able to demonstrate the special organism producing it.

The treatment will be more in the line of suggestive hints than an attempt to describe any special mode. When the pathology is understood the treatment becomes easy. Until this is accomplished our treatment must be ineffectual. Where we are not sure of our foe it is hard to choose our weapons of offence and defence.

The conditions due to calculus are simple, and demand for their cure the exercise of surgical means and methods. Remove the calculus and all diseased alveolus (this must be thoroughly done by giving several sittings); establish drainage; accomplish depletion and give the affected teeth rest, and there you have the means of a cure. In proportion to the thoroughness with which this line of treatment is carried out will be your success. No medication is necessary if this treatment be thorough. But it is as well to give the system tonics and use stimulants and antiseptics locally.

In the condition called phagedenic pericementitis the first thing is to remove any deposits that may be present as complications. After this is thoroughly accomplished it is absolutely necessary to resort to medicinal treatment. There is in well-developed cases no chance of nature effecting a cure even with the calculus entirely removed, for the exciting cause still remains. The pericementum is being disintegrated and the alveolar wall absorbed. We must remove the diseased parts with instruments. Dr. Black would not injure the gum margin, but drainage must be secured, and this must be obtained at whatever cost. Then the lacerated pockets should be washed out to remove the debris and blood-clots. For this use a solution of mercuric chloride in peroxide of hydrogen, one grain to the ounce. If there is great congestion of the gum, apply a thirty per cent. solution of chloride of zinc deep down into the pockets. Then proceed with Dr. Black's one, two, three solution.

Oil of cinnamon, 1;
Carbolic acid, cryst., 2;
Oil of gaultheria, 3.

Inject this into the pockets, once in four days, full strength and supply the patient with the same solution diluted to one-half its strength with oil of lemon or oil of anise—I prefer the former—to be used as a mouth-wash. The patient must be watched carefully, and great care must be taken to prevent a return of the irritation of the gum margin, and cleanliness must be insisted upon, with examinations continued indefinitely, for I do not believe a cure could be absolutely promised with unfavorable conditions likely to recur.—*Southern Dental Journal.*

